# Relative contribution of articular cartilage’s constitutive components to load support depending on strain rate

**DOI:** 10.1007/s10237-016-0807-0

**Published:** 2016-07-14

**Authors:** J. M. Párraga Quiroga, W. Wilson, K. Ito, C. C. van Donkelaar

**Affiliations:** Orthopaedic Biomechanics, Department of Biomedical Engineering, Eindhoven University of Technology, P.O. Box 513, 5600 MB Eindhoven, The Netherlands

## Abstract

Cartilage is considered a biphasic material in which the solid is composed of proteoglycans and collagen. In biphasic tissue, the hydraulic pressure is believed to bear most of the load under higher strain rates and its dissipation due to fluid flow determines creep and relaxation behavior. In equilibrium, hydraulic pressure is zero and load bearing is transferred to the solid matrix. The viscoelasticity of the collagen network also contributes to its time-dependent behavior, and the osmotic pressure to load bearing in equilibrium. The aim of the present study was to determine the relative contributions of hydraulic pressure, viscoelastic collagen stress, solid matrix stiffness and osmotic pressure to load carriage in cartilage under transient and equilibrium conditions. Unconfined compression experiments were simulated using a fibril-reinforced poroviscoelastic model of articular cartilage, including water, fibrillar viscoelastic collagen and non-fibrillar charged glycosaminoglycans. The relative contributions of hydraulic and osmotic pressures and stresses in the fibrillar and non-fibrillar network were evaluated in the superficial, middle and deep zone of cartilage under five different strain rates and after relaxation. Initially upon loading, the hydraulic pressure carried most of the load in all three zones. The osmotic swelling pressure carried most of the equilibrium load. In the surface zone, where the fibers were loaded in tension, the collagen network carried 20 % of the load for all strain rates. The importance of these fibers was illustrated by artificially modifying the fiber architecture, which reduced the overall stiffness of cartilage in all conditions. In conclusion, although hydraulic pressure dominates the transient behavior during cartilage loading, due to its viscoelastic nature the superficial zone collagen fibers carry a substantial part of the load under transient conditions. This becomes increasingly important with higher strain rates. The interesting and striking new insight from this study suggests that under equilibrium conditions, the swelling pressure generated by the combination of proteoglycans and collagen reinforcement accounts cartilage stiffness for more than 90 % of the loads carried by articular cartilage. This finding is different from the common thought that load is transferred from fluid to solid and is carried by the aggregate modulus of the solid. Rather, it is transformed from hydraulic to osmotic swelling pressure. These results show the importance of considering both (viscoelastic) collagen fibers as well as swelling pressure in studies of the (transient) mechanical behavior of cartilage.

## Introduction

Articular cartilage (AC) is a biphasic tissue covering the ends of bones in diarthrodial joints. Approximately 80 % of its content is fluid and the remaining 20 % solid is comprised of collagen (60–70 % of the dry weight) and proteoglycans (PGs, 20–30 % of the dry weight). AC experiences cyclic loads, which can reach up to five times body weight (Bergmann et al. 2014). Normally, the tissue can successfully carry those loads.

Common understanding is that the applied load is initially supported by the pressurized interstitial fluid (Ateshian 2009; Ateshian and Wang 1995; Oloyede and Broom 1993). This can be explained because of AC’s low permeability, which traps the fluid in the collagen-PG network becoming pressurized when the tissue is loaded. This fluid pressure is believed to shield the solid matrix from overloading (Bonnevie et al. 2012). During fast loading, it has been shown that the fluid load support exceeds 79 % (Li et al. 2014; Bonnevie et al. 2012; Park et al. 2003) leaving the remaining portion of the load to the solid. Some studies may consider the solid phase as a homogeneous isotropic elastic material (Li et al. 2014), but it is generally accepted that the true biomechanical function results from the combination of reinforcing collagen fibers and swelling of the proteoglycan-rich ground substance. The collagen network contributes to the time-dependent behavior because collagen fibers have flow-independent viscoelastic properties (Li et al. 1983; Woo et al. 1980; Hosseini et al. 2014). The exact relative contribution of the viscoelastic collagen compared to the hydraulic fluid pressure under various strain rates has not yet been elucidated. Also, because of their negative charges, proteoglycans create an osmotic pressure that participates in load sharing (Maroudas and Thomas 1970). Although osmotic swelling has been incorporated by several groups in computational models of cartilage (Lai et al. 1991; Huyghe and Janssen 1997; Sun et al. 1999; Van Loon et al. 2003), understanding the nonlinear and time-dependent contribution of osmotic pressure to the mechanical behavior of cartilage is challenging (Olsen et al. 2004).

In our composition-based cartilage model, the individual contribution of osmotic swelling pressure by proteoglycans and the effect of collagen viscoelasticity are included (Wilson et al. 2006). This allows monitoring the relative contributions of fluid pressure, osmotic pressure, collagen stress and non-fibrillar matrix stress as a function of loading magnitude, rate and duration. The aim of the present study is to demonstrate this relative load sharing under unconfined compression and indentation loading as a function of distance from the cartilage surface.

## Methods

### Material model

In our fibril-reinforced swelling poroviscoelastic model of AC (Wilson et al. 2006), two changes were implemented: The isotropic stiffness of the collagen fibers was taken into account $(\sigma _{\mathrm{f}\,\mathrm{iso}})$, and the stress in the solid phase was divided by the volumetric deformation *J*, being the total tissue stress in each integration point calculated according to Eq. 1. These adjustments allow the collagen fibers to have stiffness in compression, though very low in comparison with the stiffness in tension (Römgens et al. 2013). Because of these adjustments, material properties could not be derived from previous work, but were fitted to experimental data of unconfined compression, indentation and swelling (DiSilvestro and Suh 2001) (“Appendix”). In the updated implementation, the total stress is given as (Eq. 1):

1 $$\begin{aligned} \sigma _{\mathrm{tot}}= & {} \mu _{\mathrm{f}}{} \mathbf I +\frac{n_{\mathrm{s},0}}{J}\left( {\left( 1-\sum _{i-1}^{\mathrm{totf}} {\rho _{\mathrm{c}}^{i}}\right) \sigma _{\mathrm{nf}} +\sum _{i-1}^{\mathrm{totf}} {\rho _{\mathrm{c}}^{i}}\sigma _{\mathrm{f}\,\mathrm{iso}}^{i}}\right) \nonumber \\&-\Delta \pi \mathbf I \end{aligned}$$

where $\mu _{\mathrm{f}}$ is the fluid pressure, $\mathbf{I}$ is the unit tensor, $n_{\mathrm{s},0}$ is initial solid volume fraction, *J* is the determinant of the deformation tensor $\mathbf{F}$, $\mathrm{totf}$ is the number of fibril orientations considered at each location, $\rho _{\mathrm{c}}$ is the volume fraction of the collagen fibrils in the *i*th direction with respect to the total volume of the solid matrix, $\sigma _{\mathrm{nf}}$ is the stress in the non-fibrillar matrix, $\sigma _{\mathrm{f}\,\mathrm{iso}}$ is the stress in the collagen fiber network, and $\Delta \pi$ is the osmotic swelling pressure.

The stress in the non-fibrillar network (Eq. 2), including the shear modulus of the non-fibrillar matrix $(\mathrm{Gm}_{\mathrm{nf}})$, is:

2 $$\begin{aligned} \sigma _{\mathrm{nf}}= & {} -\frac{1}{6}\frac{{\ln }(J)}{J}\hbox {Gm}_{\mathrm{nf}}{} \mathbf{I} \left[ -1+\frac{3(J+n_{\mathrm{s},0})}{(-J+n_{\mathrm{s},0})}\right. \nonumber \\&\left. +\frac{3{\ln }(J)Jn_{\mathrm{s},0}}{(-J+n_{\mathrm{s},0})^{2}}\right] +\frac{\hbox {Gm}_{\mathrm{nf}}}{J} (\mathbf{F}\cdot \mathbf{F}^{T}-J^{2/3}{} \mathbf{I}) \end{aligned}$$

**Fig. 1:**
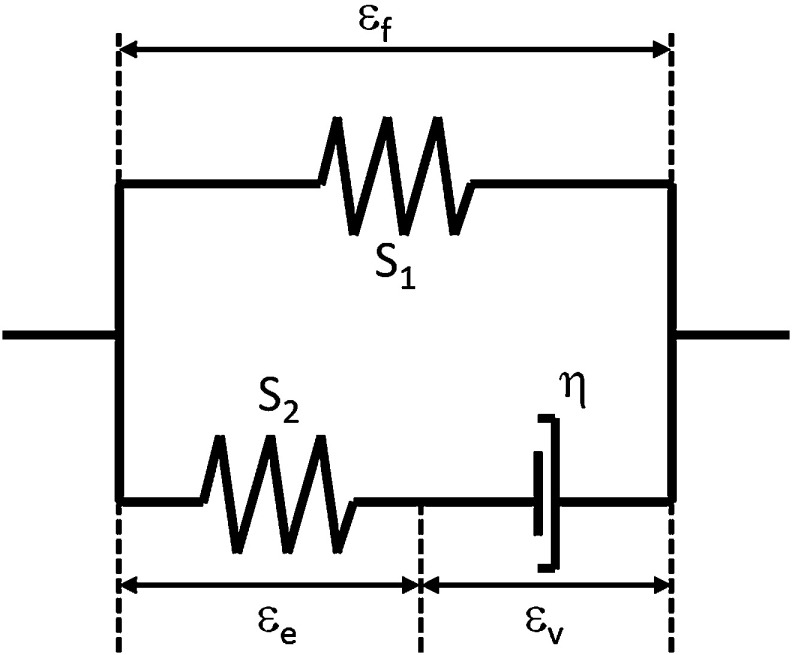
Standard linear solid model representing collagen fiber behavior, with $\varepsilon _{\mathrm{f}}$ the total fibril strain, $\varepsilon _{\mathrm{v}}$ the dashpot strain, and $\varepsilon _{\mathrm{e}}$ the strain in spring $\hbox {S}_{2}$. Where $\hbox {S}_{1}$ and $\hbox {S}_{2}$ are the stiffness of the springs and $\eta$ the viscoelastic constant of the dashpot

The equation for calculating stress in the collagen network $(\sigma _{\mathrm{f}\,\mathrm{iso}})$ is given by:

3 $$\begin{aligned} \sigma _{\mathrm{f}\,\mathrm{iso}}=\sigma _{\mathrm{f}} \vec {e}_{\mathrm{f}} \vec {e}_{\mathrm{f}}+\sigma _{\mathrm{iso}} \end{aligned}$$

where $\sigma _{\mathrm{f}}$ is described by Eqs. 4–6, $\vec {e}_{\mathrm{f}}$ is the unit vector in the current fibril direction, and $\sigma _{\mathrm{iso}}$ represents the isotropic stiffness of the fibers which was described with the same Neo-Hookean model used to describe the stress in the non-fibrillar network ($\sigma _{\mathrm{nf}}$, Eq. 2), yet with $\hbox {Gm}_{\mathrm{nf}}$ replaced by $\hbox {Gm}_{\mathrm{f}}$, representing the shear modulus of the collagen fiber network.

4 $$\begin{aligned} \sigma _{\mathrm{f}} =\frac{\lambda }{J}P_{\mathrm{f}} \vec {e}_{\mathrm{f}} \vec {e}_{\mathrm{f}} \end{aligned}$$

with $\lambda$ representing the elongation of the fibril. The total fibril stress, characterized by the nonlinear viscoelastic solid model shown in Fig. 1, is calculated as:

5 $$\begin{aligned} P_{\mathrm{f}}=P_1 +P_2 \end{aligned}$$

with $P_{1}$ and $P_{2}$ defined as:

6 $$\begin{aligned} \begin{array}{lll} P_1=E_1\left( e^{S_{1}\varepsilon _{\mathrm{f}}}-1\right) &{}\hbox {for}&{}\varepsilon _{\mathrm{f}}>0 \\ P_1=0&{}\hbox {for}&{}\varepsilon _{\mathrm{f}}\le 0 \\ P_2=E_2\left( e^{S_{1}\varepsilon _{\mathrm{e}}}-1\right) =\eta {\dot{\varepsilon }}_{\mathrm{v}}&{}\hbox {for}&{} \varepsilon _{\mathrm{e}} <0 \\ P_2=0&{}\hbox {for}&{}\varepsilon _{\mathrm{e}} \le 0 \\ \end{array} \end{aligned}$$

where $E_{1}$, $E_{2}$, $S_{1}$, $S_{2}$ and $\eta$ are material constants, $\varepsilon _{\mathrm{f}}$ is the total fibril logarithmic strain, $\varepsilon _{\mathrm{e}}$ the strain in the spring $S_{2}$, and $\varepsilon _{\mathrm{v}}$ the dashpot strain.

Two groups of collagen fibers were included (Wilson et al. 2005). One group represented the dominant or primary fibers with arcade-like orientations (Benninghoff 1925), and the second group represented a secondary non-aligned fiber network with fibers oriented in seven angular directions in 3D space ($x,\,y,\,z,\,x=y,\,y=z,\,x=z,\,x=y=z$) (Wilson et al. 2005).

The distributions of fluid, fixed charged density (FCD, derived from fluid volume and PG content) and collagen contents was implemented depth dependently according to measured data in the literature, as previously described (Wilson et al. 2006).

The material model was implemented in Abaqus 6.11-2 (Dassault Systèmes 2011).

### Simulations

Unconfined compression was simulated on a 3D representation of an osteochondral plug $(\phi =5.6\,\hbox {mm},\,\hbox {thickness}=1\,\hbox {mm})$, using both load (1 and 2 MPa) and displacement control (5, 10 and 15 %). The plug was compressed at various strain rates (ramp loading at 0.018, 0.5, 5.85, 80 and 360 [mm/min]) by an impermeable platen assuming frictionless contact. The bottom of the plug was restricted from displacements in all directions, to represent its attachment to the subchondral bone. The fluid was allowed to flow freely out of the free cartilage edges. Additionally, an indentation experiment (indenter $\phi =1\,\hbox {mm}$) with similar boundary and loading conditions was performed to reduce possible effects of adverse boundary conditions at the cut circumference of the cartilage plugs. Fluid flow was allowed on the free cartilage surface not in contact with the indenter. To evaluate time dependency, load sharing was evaluated under loading at various rates and during stress relaxation. Equilibrium was considered complete when fluid pressure dropped to zero and internal stresses reached constant values.

Each of the four components contributing to the total stress (Eq. 1) was normalized to the total stress to evaluate its individual relevance in load sharing. Because of the depth dependency present in AC for its main constitutive components (Wilson et al. 2007; Halonen et al. 2013), each component was evaluated in the superficial, middle and deep cartilage zones for each loading condition.

## Results

### FEA models

#### Unconfined compression (strain control)

**Fig. 2:**
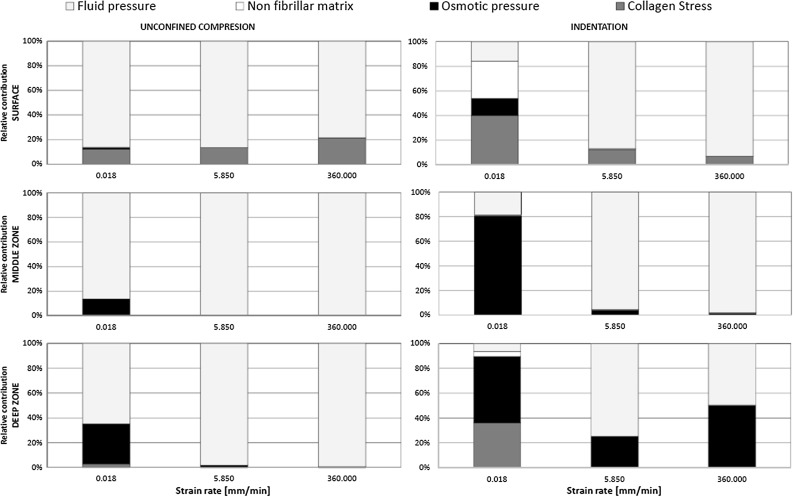
Contribution of fluid pressure, osmotic pressure, non-fibrillar matrix deviatoric stress and collagen deviatoric stress to load sharing versus loading rate at 10 % compression for elements in the surface, middle and deep zones

The maximum fluid pressure, osmotic pressure, collagen stress and non-fibrillar matrix stress were plotted against the strain rate for the three locations for 10 % strain (Fig. 2). These three locations were consistently the same for the different strain rates, and the results were monitored at the same time points. In all locations, hydraulic pressure carried most of the applied load. In the surface zone, the collagen bore up to 20 % of the load and its contribution increased with strain rate. In the middle and deep zones and for the low strain rates, the osmotic swelling carried almost 40 % of the load. In these zones, the contribution of collagen was negligible.

#### Unconfined compression (load control)

Under load-controlled compression, similar effects were observed, with a higher contribution for osmotic pressure in the deep zone. Because the plots would not add new insight to the study, the results were not shown.

#### Indentation (strain control)

Similar trends in load sharing were observed for unconfined compression and indentation (Fig. 2). However, under the indenter, the osmotic pressure in the middle and deep zones was higher than during unconfined compression.

#### Effect of superficial collagen

**Fig. 3:**
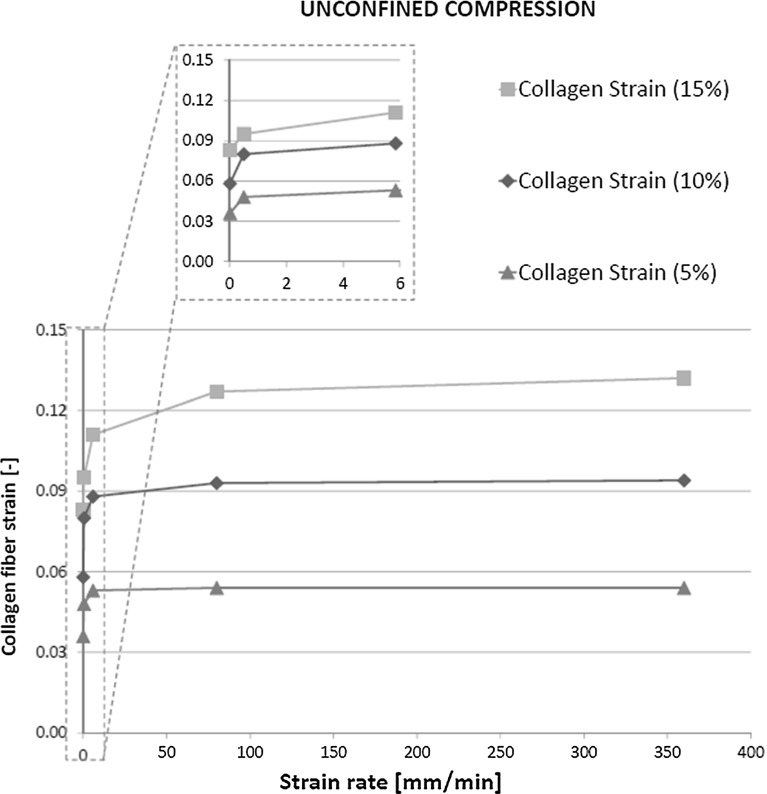
Strain in the primary collagen fibers versus loading rate for different compressive magnitudes

In unconfined compression, the tissue expanded radially and the superficial collagen fibers were strained. This deformation was dependent on the applied compressive strain, but was largely independent of the strain rate (Fig. 3).

**Fig. 4:**
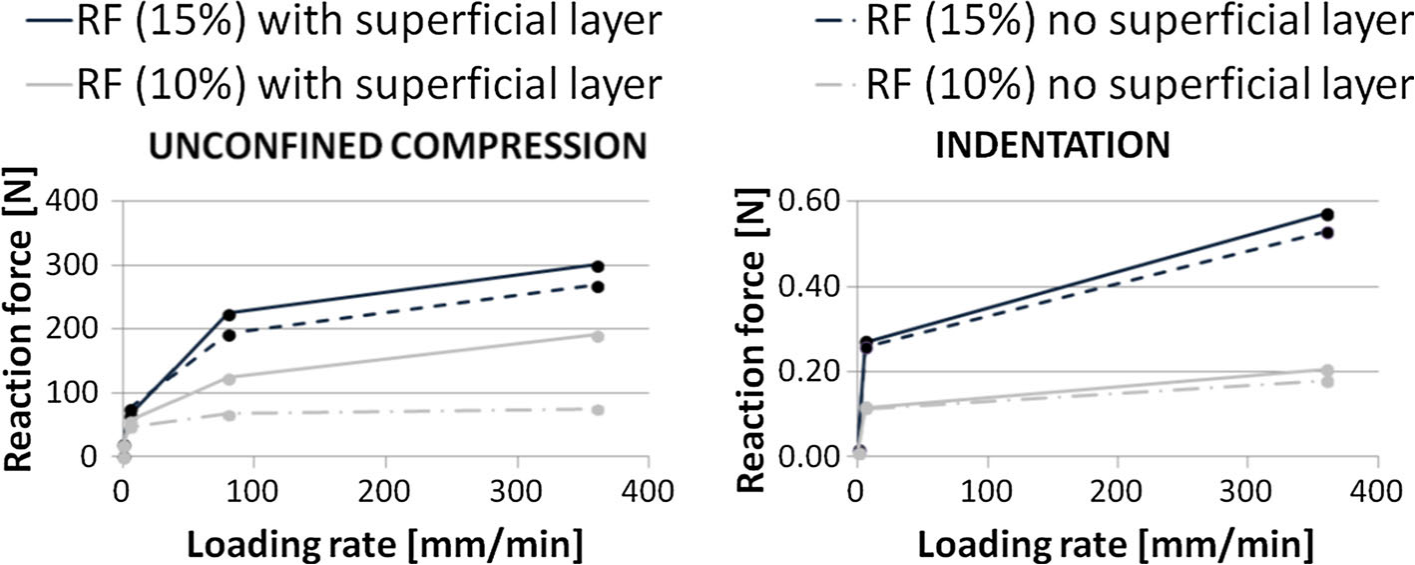
Reaction force recorded in the platen during unconfined compression (*left*) and in the indenter during indentation (*right*) for two strain magnitudes (10 and 15 %)

Because of straining, the superficial collagen fibers developed more stress than fibers in the middle and deep zones, which shortened during tissue compression. To evaluate the relevance of the 20 % load carried by the superficial collagen fibers (Fig. 2), the all primary fibrils that bend over in the middle zone and are oriented radially in the superficial layer, were replaced by primary fibers that are perpendicular to the surface, aligning with those in the deep zone. This significantly reduced the reaction force for the same applied strain, i.e., cartilage behaved weaker as a consequence of the change in collagen orientation. This effect was loading rate dependent. The effect of surface collagen orientation during indentation was less pronounced than for unconfined compression (Fig. 4).

#### Stress relaxation in unconfined compression (strain control)

**Fig. 5:**
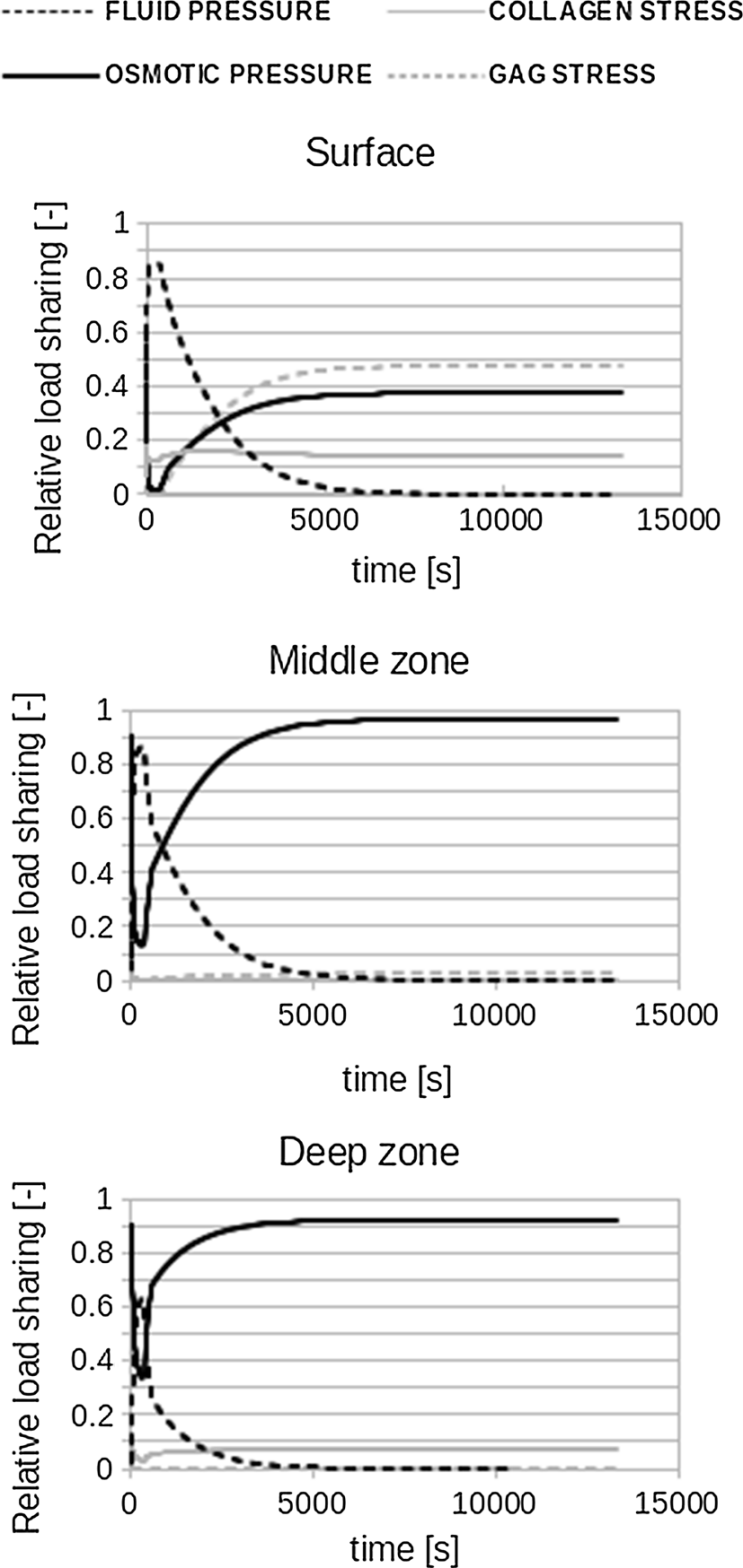
Contribution of the four main components to load sharing during stress relaxation under 10 % strain. Data are similar for 15 % strain

During stress relaxation, the load support was shifted from hydraulic to osmotic pressure, which dominated the compressive properties in the middle and deep zones (Fig. 5). The solid (GAG and collagen) carried considerable load during equilibrium in the superficial zone.

## Discussion

In agreement with common understanding of biphasic mechanics, fluid pressure initially takes most of the load applied on cartilage. This is regardless of the loading type (unconfined compression vs indentation), rate or magnitude. This study shows that the second most important constituent for initial load bearing is the collagen network in the superficial zone. During unconfined compression, this may take 20 % of the total load in this zone. Without radial alignment of collagen in the superficial zone, the overall stiffness of cartilage is reduced up to 50 %, depending on strain rate and magnitude (Fig. 4). These effects are more pronounced during unconfined compression than during indentation. After few minutes of sustained loading, the contribution of hydraulic pressure decreases and osmotic pressure starts to dominate load carriage in cartilage, taking approximately 95 % of the total load throughout the middle and deep zones in equilibrium. This is in contrast with common thought that during stress relaxation, load is slowly transferred from the fluid to the solid phase. This is true for load transfer in biphasic tissues that do not have osmotic swelling capacity. However, in cartilage with strong osmotic swelling potential, the solid is hardly loaded even in equilibrium. Rather, the load is transferred from hydraulic to osmotic pressure in the fluid.

The relevance of an intact superficial collagen network for AC mechanics has been shown previously under equilibrium conditions (Hosseini et al. 2014; Bevill et al. 2010; Thambyah et al. 2009). The present study demonstrated that in addition, the superficial collagen contributed significantly to instantaneous cartilage behavior at different strain rates (Figs. 2 and 4). Interestingly, despite this strain rate-dependent effect, the superficial collagen strain only increased for very low strain rates. At modest strain rates, the fiber strains reached a limit (Fig. 3), and their contribution to load bearing did not increase further (Fig. 2). The explanation may be that at low strain rates, fluid may internally flow or even leave the cartilage. At strain rates above a particular strain rate, fluid will not have time to redistribute within the tissue and the cartilage deforms as an incompressible material. Under such conditions, the strain distribution within the tissue may have become less dependent on the strain rate (Fig. 3). However, this does not mean that the maximum collagen strain has been reached; when more strain is applied on the tissue, the tissue deforms more and the strain in the superficial collagen fibers follows tissue deformation, reaching a higher strain level (Fig. 3). This is different under indentation loading, where total tissue deformation is less significant, collagen strain does not reach a limit, and therefore, its effect on load bearing continues to change with strain rate (Fig. 2).

Based on the above evaluation, we may speculate that collagen damage is closely related to large forces/deformations than to strain rate; the dependence on strain rate might only hold for rates below approximately 80 mm/min. Whether such speculation holds true remains to be determined in future work. Interestingly, at 10 % tissue compression, the superficial collagen strain is 9 %, while it reaches 13 % when the tissue is compressed 15 %. Although the damaging strain limit for collagen type II is unknown, based on experimental work with other collagen types, it has been previously assumed that fibers may start degenerating at strains in the order of about 8 % (Hosseini et al. 2014). Thus, this would indicate that 10 % unconfined compression would not result in significant tissue fibrillation, whereas 15 % compression would. Indeed, it was recently determined that cartilage compressive strain reaches 15 % after meniscectomy, and this condition is known to induce cartilage damage (Párraga Quiroga et al. 2015).

With regard to damage development, it has been speculated that the initial hydraulic pressure in the fluid may protect the matrix from becoming damaged. However, under sustained loading, it was expected that fluid pressure would cease and load was transferred to the solid matrix, which would then become susceptible for damage. Interestingly, the present study shows that load is not transferred to the solid matrix, but rather to osmotic fluid pressure, which even carries 95 % of the load. Thus, cartilage properties during unconfined compression seems to be dominated by a fluid pressure both initially and during sustained loading, yet of a different nature. This finding may shed a different light on the interpretation of past studies to cartilage mechanics both numerical and experimental. Interpretations related to load transfer from fluid to solid under sustained loading of cartilage are to be reconsidered, i.e., if no osmotic swelling was considered before, the entire load initially carried by the fluid will necessarily be transferred to the solid during transient behavior. However, this study shows that the solid does not take most of the load, but the osmotic pressure generated due to the proteoglycan content does. This study also emphasizes the need for the use of models that include both osmotic swelling and fiber reinforcement to capture the intrinsic mechanical behavior of cartilage.

Furthermore, the current finding about the importance of including osmotic swelling suggests that the behavior of the material when loaded at low strain rates depends largely on the osmotic swelling, which in turn depends on the PG content. As strain rates increase, this shifts such that behavior depends mainly on the water content. Although the amount of collagen does not seem to have a large influence on AC mechanical behavior in Fig. 5 of this study, this is not true. It is only because of the presence of the collagen fibers that the tissue is able to develop a large osmotic pressure. Without collagen, the tissue would swell dramatically, which would then result in a strong reduction in the osmotic pressure.

**Fig. 6:**
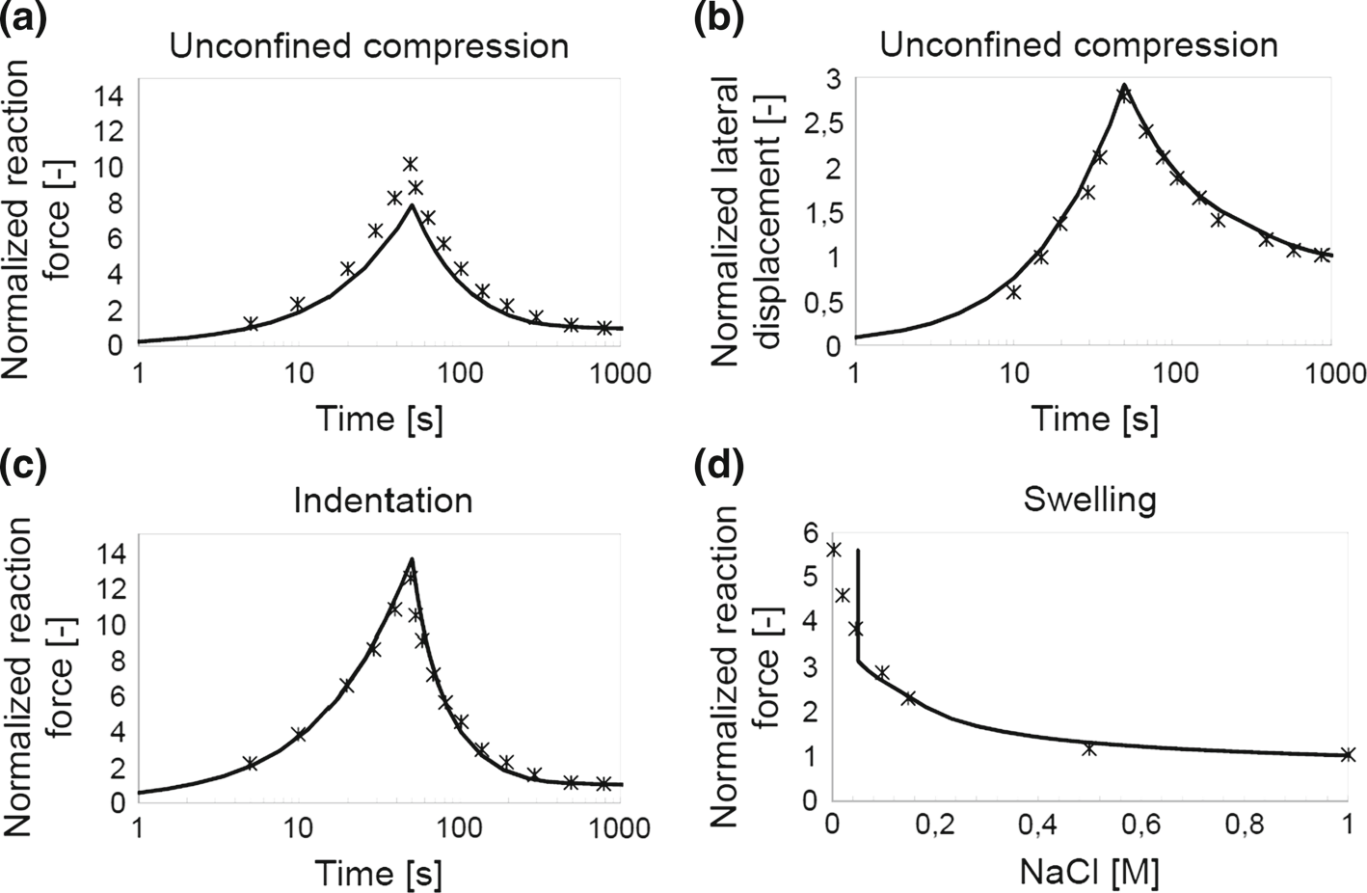
**a**, **c** and **d** Reaction force normalized to experimental data. **b** Lateral displacement during unconfined compression. *Line* $=$ numerical fit; *stars* $=$ experimental data (DiSilvestro and Suh 2001)

Using advanced material models that account for osmotic pressure and fluid, however, also comes with limitations. The present study uses a 3D model with an idealized shape. Using a more realistic joint shape in combination with a sophisticated material description is challenging and computationally expensive (Meng et al. 2014). Simpler material models have been used to compute mechanical conditions in more realistic 3D geometries. Simplifications may, for instance, involve neglecting the osmotic pressure (Jones et al. 2016; Mononen et al. 2016), which this study shows to be essential for load bearing. As highlighted by Kazemi et al. (2013), due to smoothing algorithms to obtain the geometry from images, the obtained cartilage layers are not completely representative of the actual cartilage. Minor changes in the thickness can produce different contact. Consequently, the mechanical conditions are less accurate and this may affect also outcome parameters such as contact pressure patterns. A direct comparison between model predictions would be an interesting future study. Also, larger 3D models require coarser meshes to reduce the total number of elements. This may affect mesh quality and may not allow to distinguish appropriately between deep, middle and superficial zones in the cartilage, with similar inaccuracies in mechanical conditions as a result. Thus, there is always a trade-off between using realistic geometries and accurate computations from the tissue material point of view, and the various approaches provide additional insights in cartilage and joint mechanics. In the present study, an unconfined compression experiment of an osteochondral explant was simulated. Such experiments are a commonly used alternative to testing the AC in the full knee joint and are considered to be closer to physiologic conditions than confined compression (Park et al. 2003). Thus, using this for our study to fundamental cartilage mechanics in combination with effects of indentation is appropriate. Nevertheless, it would be interesting to compare our findings of the effect of collagen orientation in the superficial surface against models that use more realistic 3D geometries (Jones et al. 2016; Mononen et al. 2016). The latter studies do not consider osmotic pressure. However, the present study shows that osmotic pressure plays a lesser role in the superficial zone than in the deep zone (Fig. 2), suggesting that the effect of collagen orientation in the superficial zone may be relatively independent of osmotic pressure. A direct comparison between models could be of interest in the future.

It should be noted that the above discussion on conditions which might result in collagen damage is speculative. A true cartilage damage model could assist in answering the question whether damage would indeed develop under these conditions. Development and experimental validation of such model is ongoing (Hosseini et al. 2014).

In conclusion, it is shown that the stress in the non-fibrillar proteoglycan-rich matrix does not contribute much to load bearing. Rather, it generates an osmotic pressure that contributes to 95 % of the load bearing under slow strain rates or in equilibrium. In agreement with common understanding, this study shows that fluid pressure dominates load bearing immediately upon cartilage loading. Finally, it shows a significant contribution for superficial collagen fibers at faster strain rates.

## Appendix

The updated AC numerical model was fitted to experimental data from DiSilvestro and Suh (2001), Fig. 6. For more details about the fitting procedures and the parameters fitted, the reader is referred to Wilson et al. (2005). The final fits and associated parameters were:

$$\begin{aligned} \hbox {Gm}_{\mathrm{nf}}= & {} 0.7722;\quad \hbox {Gm}_{\mathrm{f}}=0.01144;\quad E_{1}=4.362;\\ S_{1}= & {} 14.39;\quad E_{2}=20.25;\quad S_{2}=43.96;\quad \eta =153200. \end{aligned}$$

## References

[CR1] Ateshian GA, Wang H (1995). A theoretical solution for the frictionless rolling contact of cylindrical biphasic articular cartilage layers. J Biomech.

[CR2] Ateshian GA (2009). The role of interstitial fluid pressurization in articular cartilage lubrication. J Biomech.

[CR3] Bank RA, Soudry M, Maroudas A, Mizrahi J, TeKoppele JM (2000). The increased swelling and instantaneous deformation of osteoarthritic cartilage is highly correlated with collagen degradation. Arthritis Rheum.

[CR4] Benninghoff A (1925). Form und bau der gelenkknorpel in ihren beziehungen zur function. II. Der aufbau des gelenkknorples in seinen beziehungen zur function. Z Zellforsch Mikrosk Anat.

[CR5] Bergmann G, Bender A, Graichen F, Dymke J, Rohlmann A, Trepczynski A, Heller MO, Kutzner I (2014). Standarized loads acting in knee implants. PLoS One.

[CR6] Bevill SL, Thambyah A, Broom ND (2010). New insights into the role of the superficial tangential zone in influencing the microstructural response of articular cartilage to compression. Osteoarthr Cartil.

[CR7] Bonnevie ED, Baro VJ, Wang L, Burris DL (2012). Fluid load support during localized indentation of cartilage with a spherical probe. J Biomech.

[CR8] Buckwalter JA, Mankin HJ (1998). Articular cartilage: degeneration and osteoarthritis, repair, regeneration, and transplantation. Instr Course Lect.

[CR9] DiSilvestro MR, Suh JK (2001). A cross-validation of the biphasic poroviscoelastic model of articular cartilage in unconfined compression, indentation, and confined compression. J Biomech.

[CR10] Felson DT, Zhang Y, Hannan MT, Naimark A, Weissman B, Aliabadi P, Levy D (1997). Risk factors for incident radiographic knee osteoarthritis in the elderly: the Framingham Study. Arthritis Rheum.

[CR11] Grenier S, Bhargava MM, Torzilli PA (2014). An in vitro model for the pathological degradation of articular cartilage in osteoarthritis. J Biomech.

[CR12] Halonen KS, Mononen ME, Jurvelin JS, Töyräs J, Korhonen RK (2013). Importance of depth-wise distribution of collagen and proteoglycans in articular cartilage - a 3D finite element study of stresses and strains in human knee joint. J Biomech.

[CR13] Hosseini A, van de Velde SK, Kozanek M, Gill TJ, Grodzinsky AJ, Rubash HE, Li G (2010). In-vivo time-dependent articular cartilage contact behavior of the tibiofemoral joint. Osteoarthr Cartil.

[CR14] Hosseini SM, Wilson W, Ito K, van Donkelaar CC (2014). A numerical model to study mechanically induced initiation and progression of damage in articular cartilage. Osteoarthr Cartil.

[CR15] Huyghe JM, Janssen JD (1997). Quadriphasic theory of swelling incompressible porous media. Int J Eng Sci.

[CR16] Hwang WS, Li B, Jin LH, Ngo K, Schachar NS, Hughes GN (1992). Collagen fibril structure of normal, aging, and osteoarthritic cartilage. J Pathol.

[CR17] Jones B, Hung CT, Ateshian G (2016). Biphasic analysis of cartilage stresses in the patellofemoral joint. J Knee Surg.

[CR18] Kazemi M, Dabiri Y, Li LP (2013). Recent advances in computational mechanics of the human knee joint. Comput Math Methods Med.

[CR19] Lai WM, Hou JS, Mow VC (1991). A triphasic theory for the swelling and deformation behaviours of articular cartilage. J Biomech Eng.

[CR20] Li J, Hua X, Jin Z, Fisher F, Wilcox R (2014) Biphasic investigation of contact mechanics in natural human hips during activities. Proc Inst Mech Eng H 228:556–563 10.1177/0954411914537617 PMC4361357 24898443

[CR21] Li JT, Armstrong CG, Mow VC (1983). Effect of strain rate on mechanical properties of articular cartilage in tension. Proc Biomech Symp Trans ASME.

[CR22] Maroudas A, Thomas H (1970). A simple physicochemical micromethod for determining fixed anionic groups in connective tissue. Biochim Biophys Acta.

[CR23] McDevitt C, Gilberston E, Muir H (1977). An experimental model of osteoarthritis; early morphological and biochemical changes. J Bone Joint Surg Br.

[CR24] Meng QE, Jin ZM, Wilcox R, Fisher J (2014). Computational investigation of the time-dependent contact behaviour of the human tibiofemoral joint under body weight. Proc Inst Mech Eng H.

[CR25] Mononen ME, Tanska P, Isaksson H, Korhonen RK (2016). A novel method to simulate the progression of collagen degeneration of cartilage in the knee: data from the osteoarthritis initiative. Sci Rep.

[CR26] Oloyede A, Broom N (1993). Stress-sharing between the fluid and solid components of articular cartilage under varying rates of compression. Connect Tissue Res.

[CR27] Olsen S, Oloyede A, Adam C (2004). A finite element formulation and program to study transient swelling and load-carriage in healthy and degenerate articular cartilage. Comput Methods Biomech Biomed Eng.

[CR28] Park S, Krishnan R, Nicoll SB, Ateshian GA (2003). Cartilage interstitial fluid load support in unconfined compression. J Biomech.

[CR29] Párraga Quiroga JM, Ito K, van Donkelaar CC (2015). Meniscus replacement: influence of geometrical mismatches on chondroprotective capabilities. J Biomech.

[CR30] Römgens AM, van Donkelaar CC, Ito K (2013). Contribution of collagen compressive stiffness of cartilaginous tissues. Biomech Model Mechanobiol.

[CR31] Setton LA, Zhu W, Mow VC (1993). The biphasic poroviscoelastic behavior of articular cartilage: role of the surface zone in governing the compressive behavior. J Biomech.

[CR32] Sun DN, Gu WY, Guo XE, Lai WM, Mow VC (1999). A mixed finite element formulation of triphasic mechano-electrochemical theory for charged, hydrated biological soft tissues. Int J Numer Methods Eng.

[CR33] Thambyah A, Zhao L, Broom ND (2009). Microstructural response and fluid flow mechanisms in cartilage loading: new insights using a novel indentation method. J Strain Anal Eng Des.

[CR34] van Loon R, Huyghe JMRJ, Wijlaars MW, Baaijens FPT (2003). 3D FE implementation of an incompressible quadriphasic mixture model. Int J Numer Method Biomed Eng.

[CR35] Venn M, Maroudas A (1977). Chemical composition and swelling of normal and osteoarthrotic femoral head cartilage. Ann Rheum Dis.

[CR36] Wilson W, van Donkelaar CC, van Rietbergen B, Huiskes R (2005). A fibril-reinforced poroviscoelastic swelling (FPVES) model for articular cartilage. J Biomech.

[CR37] Wilson W, Huyghe JM, van Donkelaar CC (2006). A composition-based cartilage model for the assessment of compositional changes during cartilage damage and adaptation. Osteoarthr Cartil.

[CR38] Wilson W, Huyghe JM, van Donkelaar CC (2007). Depth-dependent compressive equilibrium properties of articular cartilage explained by its composition. Biomech Model Mechanobiol.

[CR39] Woo SLY, Simon BR, Kuei SC, Akeson WH (1980). Quasi-linear viscoelastic properties of normal articular cartilage. J Biomech Eng.

